# Supplementary material for: Exposure Assessment of Cadmium in Female Farmers in Cadmium-Polluted Areas in Northern Japan
Source: Toxics. 2020 Jun 17;8(2):44. doi: 10.3390/toxics8020044 (PMC7356160; doi:10.3390/toxics8020044)
Supplement: Supplementary file 1 [file toxics-08-00044-s001.pdf]

Supplementary Materials: Exposure Assessment of Cadmium in Female Farmers in Cadmium-Polluted Areas in Northern Japan

Hyogo Horiguchi, Etsuko Oguma, Satoshi Sasaki, Kayoko Miyamoto, Yoko Hosoi, Akira Ono, and Fujio Kayama

Table S1. Annual trend of rates of rice with a cadmium concentration above the safety standard (0.4 mg/kg) in the intensive inspections targeted for 3 cadmium-polluted areas in Akita prefecture, Japan, around the start of the flooding of paddy fields (2002). These inspections were prosecuted based on the Anti-Farm Soil Pollution Law of Japan, and the results are open to the public (Akita Prefect. Dep. of Agriculture, Forestry and Fisheries). Data are presented as percentages of rice with a cadmium concentration above the safety standard along with (numbers of rice with a cadmium concentration above the safety standard/total numbers of inspected rice).

Areas	2000	2001	2002	2003	2004
Odate	25.0%<br>(3/12)	13.5%<br>(5/37)	14.3%<br>(3/21)	13.3%<br>(2/15)	0%<br>(0/8)
Kazuno	37.5%<br>(12/32)	39.7%<br>(27/68)	11.8%<br>(8/68)	3.1%<br>(2/65)	1.4%<br>(1/71)
Kosaka	38.5%<br>(5/13)	37.5%<br>(6/16)	12.5%<br>(2/16)	0%<br>(0/17)	0%<br>(0/24)
